# Genome-Wide Identification of *MYB* Transcription Factors and Screening of Members Involved in Stress Response in *Actinidia*

**DOI:** 10.3390/ijms23042323

**Published:** 2022-02-19

**Authors:** Hui Xia, Xinling Liu, Zhiyi Lin, Xuefeng Zhang, Meijing Wu, Tong Wang, Honghong Deng, Jin Wang, Lijin Lin, Qunxian Deng, Xiulan Lv, Kunfu Xu, Dong Liang

**Affiliations:** College of Horticulture, Sichuan Agricultural University, Chengdu 611130, China; xiahui@sicau.edu.cn (H.X.); 2020205016@stu.sicau.edu.cn (X.L.); 2020205019@stu.sicau.edu.cn (Z.L.); 2021205010@stu.sicau.edu.cn (X.Z.); 2021205015@stu.sicau.edu.cn (M.W.); 2021205013@stu.sicau.edu.cn (T.W.); 14561@sicau.edu.cn (H.D.); 14224@sicau.edu.cn (J.W.); llj800924@sicau.edu.cn (L.L.); 10661@sicau.edu.cn (Q.D.); 10998@sicau.edu.cn (X.L.); 71389@sicau.edu.cn (K.X.)

**Keywords:** kiwifruit, *MYB* transcription factor, genome-wide identification, stress response, lignin biosynthesis

## Abstract

*MYB* transcription factors (TFs) play an active role in plant responses to abiotic stresses, but they have not been systematically studied in kiwifruit (*Actinidia chinensis*). In this study, 181 *AcMYB* TFs were identified from the kiwifruit genome, unevenly distributed on 29 chromosomes. The high proportion (97.53%) of segmental duplication events (Ka/Ks values less than 1) indicated that *AcMYB* TFs underwent strong purification selection during evolution. According to the conservative structure, 91 *AcR2R3-MYB* TFs could be divided into 34 subgroups. A combination of transcriptomic data under drought and high temperature from four *AcMYB* TFs (*AcMYB2*, *AcMYB60*, *AcMYB61* and *AcMYB102*) was screened out in response to stress and involvement in the phenylpropanoid pathway. They were highly correlated with the expression of genes related to lignin biosynthesis. qRT-PCR analysis showed that they were highly correlated with the expression of genes related to lignin biosynthesis in different tissues or under stress, which was consistent with the results of lignin fluorescence detection. The above results laid a foundation for further clarifying the role of *MYB* in stress.

## 1. Introduction

*MYB* is one of the largest transcription factor (TF) families in plants, and it is characterized by containing the *MYB* domain [1]. According to the number of repetitions of this domain and its similarity to prototype repetitions, it was divided into four categories, namely, *4R-MYB*, *R1R2R3-MYB*/*3R-MYB*, *R2R3-MYB* and *1R-MYB/MYB-Related* [2]. Among them, *R2R3-MYB* TF is one of the most abundant MYB proteins in plants, with two *MYB* domains at the N-terminal and most transcriptional activation domains at the C-terminal. It has a wide range of effects in plants, such as regulation of cell morphogenesis, meristem formation, hormone response, secondary metabolism and environmental stress response [3]. Many *R2R3-MYB* TFs have been reported to be involved in stress response, such as *AtMYB12* [4], *AtMYB20* [5], *AtMYB75* [6] and so on. Notably, the expression of these *R2R3-MYB* TFs is often accompanied by the accumulation of phenylpropanoid, such as flavonoid, anthocyanin and lignin when induced by stress [4,5,6]. Moreover, the allocation of metabolic flux among the three is one of the means for plants to respond to changes in the external environment [6]. In particular, the lignin biosynthesis pathway, as the center of biosynthesis [7], is more significantly affected by the other two.

Kiwifruit belongs to *Actinidiaceae* (*Actinidia* Lindl.). Because of its large and thin leaves, thick stem pith and thick shallow roots, compared with other fruit trees, it is more sensitive to stress [8]. Fortunately, its fruit is rich in phenylpropanoid, such as anthocyanin and flavonoid, which can help plants resist stress [4,6]. However, more attention has been paid to the enrichment of fruit color by the two in the existing studies [9,10] without noticing its response to environmental stress, as well as the relationship between benzene and propane metabolism and stress response, especially lignin, which is the center of biosynthesis [7]. Although *MYB* TFs have been reported to be involved in the regulation of lignin biosynthesis [11,12], most of the existing studies related to lignin in kiwifruit have focused on its impact on post-harvest quality [13] or its prevention and treatment of cancer [14], neglecting the fact that its accumulation could alleviate the poor drought and moisture tolerance of kiwifruit [15]. This study aims to provide a reference for solving such problems, focusing on the identified 181 *AcMYB* TFs. Their chromosome distribution, gene duplication events, protein evolution, gene structure and conservative domain were analyzed. Moreover, *AcMYB* members’ responses to abiotic stresses were screened out based on the transcriptomic data. Quantitative real-time PCR (qRT-PCR) was used to explore the spatial and temporal expression differences and co-expression effects in different tissues under different stresses. The results will provide a reference for further study on the role of *MYB* TFs in stress.

## 2. Results

### 2.1. Identification and Chromosomal Distribution of MYBs TFs in Kiwifruit

According to the predictions made by Plant TFDB database, 138 *AcMYB-related* TFs and 97 *AcMYB* TFs were preliminarily identified from the kiwifruit genome. Then, TBtools was combined with NCBI bidirectional blast and hmmer 3.0 for retrieval, and 181 *AcMYB* TF genes were finally obtained, including 87 *AcMYB-related* sequences, 91 *AcR2R3-MYB* sequences and 3 *3R-MYB* sequences (*Achn093361*/*Achn191781* and *Achn365361*/*Achn029591* as redundant genes with completely consistent sequences). The basic physicochemical properties are shown in Table 1 (Tables S2–S4).

**Figure 1:**
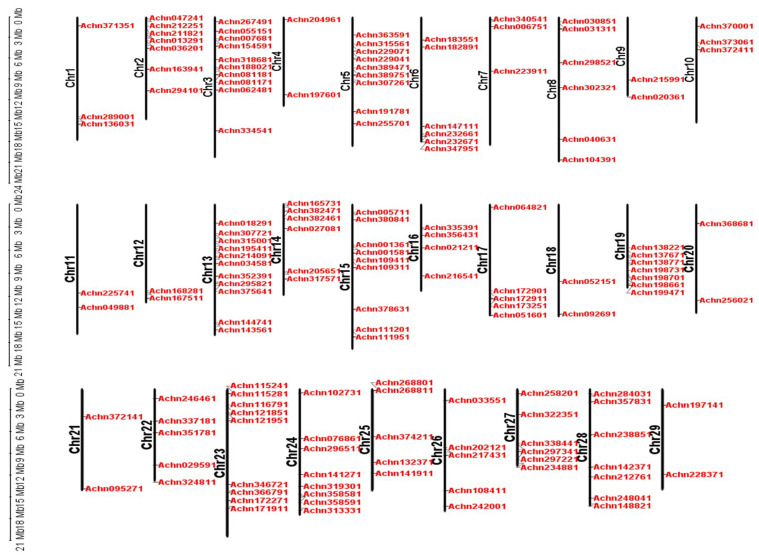
Chromosome distribution of *AcMYB* TFs in kiwifruit.

Genomic localization analysis showed that all 29 chromosomes had *AcMYB* TFs distribution. Chromosome 13 had the highest number of *AcMYB* TFs (11). A minimum of two *AcMYB* TFs was detected on chromosomes 4, 9, 11, 12, 18, 20, 21 and 29 (Figure 1).

### 2.2. Phylogeny Analysis of AcMYB TFs

**Figure 2:**
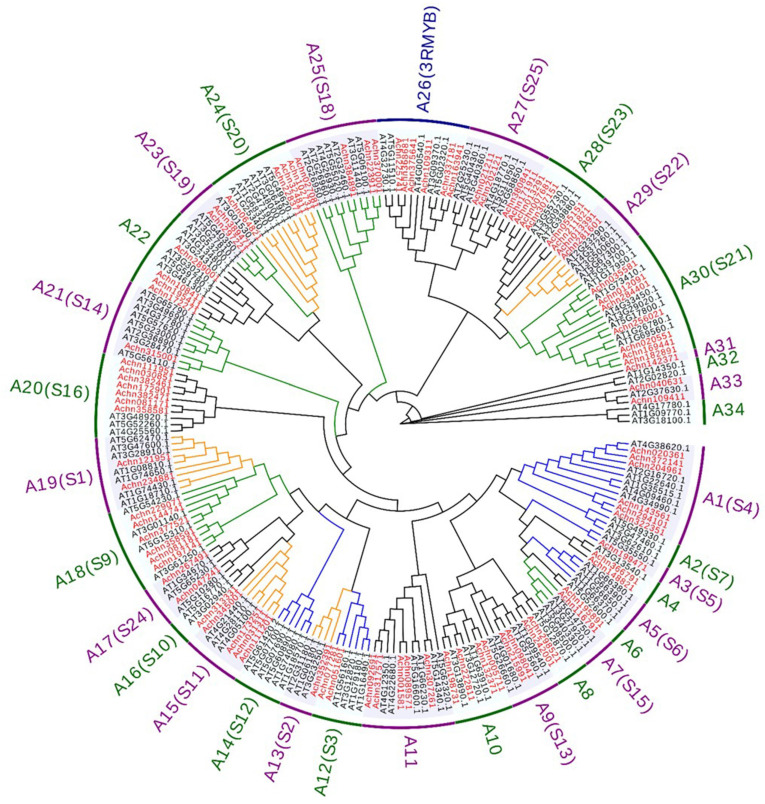
Phylogenetic analysis of *R2R3-MYB*, *Arabidopsis thaliana* classification S1–S25 with reference to Dubos [2], with red as *AcMYBs* and black as *AtMYBs*, orange line as stress response-related *MYBs*, blue line as metabolism-related *MYBs* and green line as growth-related *MYBs*.

To elucidate the phylogenetic relationship of the *AcR2R3-MYB* TFs in kiwifruit, 91 *AcMYBs* and 131 *AtMYBs* members were subjected to phylogenetic tree construction (*Arabidopsis thaliana* classification S1–S25 with reference to Dubos [2]). They were found to be divided into 34 subgroups, with all members of the A26 subgroup being *3R-MYBs* (Figure 2). Previous studies have shown that the S1 (A19) [16], S2 (A13) [17], S11 (A15) [18], S20 (A24) [19] and S22 (A29) [20] subgroups in *Arabidopsis thaliana* are involved in the regulation of resistance to biotic and abiotic stresses. The S3 (A12) [21], S4 (A1), S5 (A3), S6 (A5) [22,23], S7 (A2) [24] and S12 (A14) subgroups participated in the regulation of primary and secondary metabolism in *Arabidopsis thaliana*. The S9 (A18), S14 (A21) [25,26], S15 (A7) [27,28], S18 (A25), S19 (A23) [29,30] and S21 (A30) subgroups affect the growth and development process of *Arabidopsis thaliana*. The gene functions of the subgroups S13 (A9), S16 (A20), S23 (A28), S24 (A17) and S25 (A27) have not been completely analyzed, and they are temporarily classified into a category of function to be determined. Notably, although the gene function of the S13 subgroups has not been fully resolved, it has also been reported that it could help plants resist drought stress [31].

### 2.3. Conservative Motif and Gene Structure Analysis of AcR2-MYB and AcR3-MYB TFs

**Figure 3:**
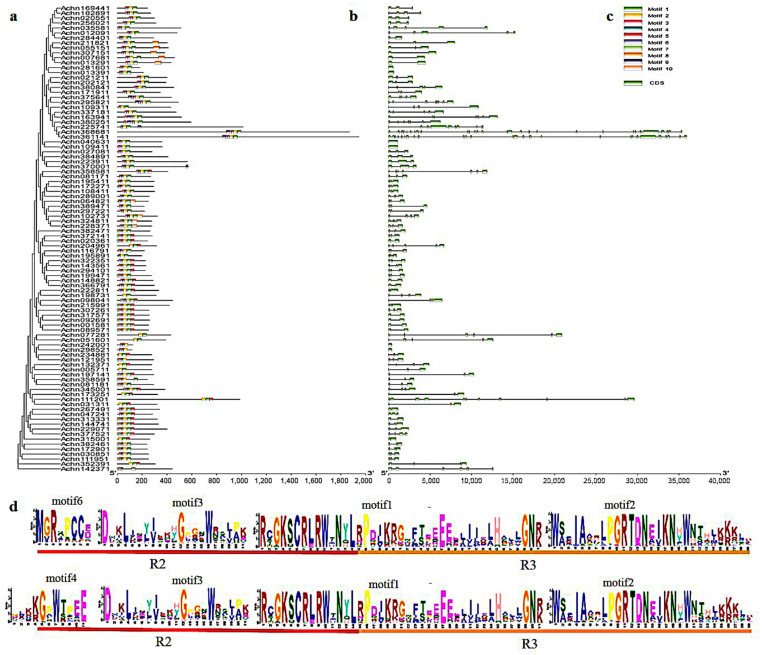
Subgroups of *AcR2-MYB* and *AcR3-MYB* genes in kiwifruit (**a**) protein evolution analysis, (**b**) conservative motif analysis, (**c**) gene structure analysis and (**d**) conservative domain sequence.

Using MEME to identify the conservative motifs in the protein sequences of *AcR2-MYBs* and *AcR3-MYBs* (R2R3-MYBs and 3R-MYBs protein sequences) in kiwifruit, it was found that most AcMYBs proteins had motif4/motif 6, motif 3, motif1 and motif 2, which formed the *R2R3-repeat* (Figure 3d). *R2-MYB* and *R3-MYB* are homologous to the subfamily *R2R3-repeat*, each of which contains a highly conservative tryptophan residue (W) in its repeating domain, and these motifs occur in almost all genes. Notably, *Achn375641* and *Achn295821*, *Achn163941* and *Achn380251*, *Achn361141* and *Achn368681*, all contain motif 9 (including the moiety R1-reapeat), which can be classified as *3R-MYB*. Based on the information of gene structure and phylogenetic analysis, we also found that *AcMYBs* in different subgroups contained different numbers of exons. The genes in the same subgroup showed highly similar gene structure and conservative motifs (Figure 3a–c).

### 2.4. Gene Duplication and Synteny Analysis of AcMYB TFs in Kiwifruit

**Figure 4:**
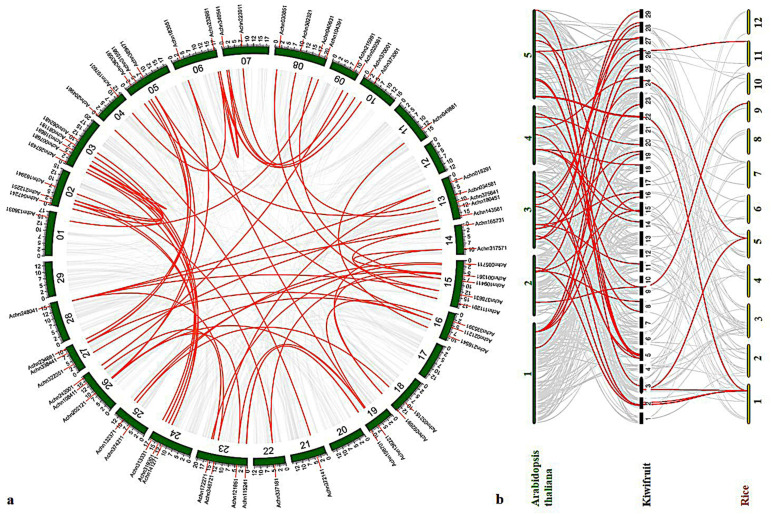
Collinearity analysis of *AcMYBs* TF in kiwifruit. (**a**) Collinearity analysis and gene duplication in kiwifruit genome. The gray line represents all autosomal blocks in kiwifruit genome, the red line represents the *AcMYB* gene duplication events, and the *AcMYB* gene is represented by a vertical red line. (**b**) Collinearity analysis of kiwifruit and two model plants; the green chromosome is *Arabidopsis thaliana*, and the yellow chromosome is rice.

An estimation of gene duplication events in kiwifruit genome by collinearity analysis revealed that two tandem duplication events, located in Chr6 and Chr14, respectively, occurred in its evolution. A total of 78 segmental duplication events, including 99 genes, were distributed across the remaining 25 chromosomes, except for Chr12/17/20/29 (Figure 4a), and an ultrahigh proportion (97.53%) of segmental duplication was identified. At the same time, the Ka/Ks values of all collinearity pairs were less than 1 (Table S5), indicating that *AcMYB* TFs in kiwifruit underwent strong purification selection during evolution.

In order to further deduce the evolutionary mechanism of *AcMYBs* in kiwifruit, a comparative syntenic map of kiwifruit and two model plants, namely, *Arabidopsis thaliana* and rice, was constructed. The results showed that there were 39 orthologous genes between kiwifruit and *Arabidopsis thaliana* (Table S6) and only 9 orthologous genes between kiwifruit and rice (Table S7). Among them, four *AcMYBs* could be found in a syntenic relationship with both *Arabidopsis thaliana* and rice, suggesting that it might exist before the differentiation of monocotyledonous and dicotyledonous plants.

### 2.5. Expression Analysis Based on Transcriptomic Data

**Figure 5:**
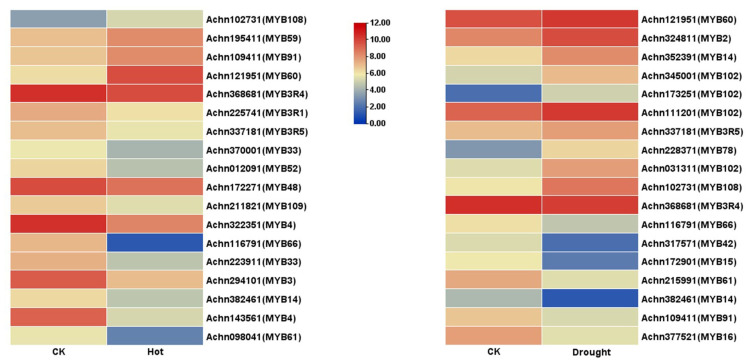
Heatmap of *AcR2R3-MYB* TFs’ transcriptional profiles in kiwifruit under drought and high-temperature stress.

Transcriptional abundance of *AcR2R3-MYB* TFs members was extracted from the kiwifruit transcriptomic data under drought and high-temperature stresses previously sequenced by our group [32,33] (Tables S8 and S9). Among them, 31 members did not detect transcriptional abundance in the control or drought stress treatment group, and 26 members did not detect transcriptional abundance in the control or high-temperature stress treatment group. After the low-quality reading (padj > 0.05) was removed, the heatmap (Figure 5) was generated with corresponding FPKM values of the control or stress treatment group using the TBtools. Among them, there were 14 down-regulated *AcMYBs* in the high-temperature treatment group, where |log_2_fold change| > 2 was down-regulated by *AcMYB3*, *AcMYB4*, *AcMYB61* and *AcMYB66*; 4 up-regulated *AcMYBs*, of which, *AcMYB60* up-regulated |log_2_fold change| > 2. In the drought treatment group, there were 8 down-regulated *AcMYBs*, among which *AcMYB14*, *AcMYB15*, *AcMYB16*, *AcMYB42* and *AcMYB61* down-regulated |log_2_fold change| > 2; 10 up-regulated *AcMYBs*, of which |log_2_fold change| > 1.5 for *AcMYB2*, *AcMYB78*, *AcMYB102* and *AcMYB108* were up-regulated.

### 2.6. Screening of Stress-Responsive AcMYB TFs and Prediction Regulatory Network

Since homologous genes usually have similar functions, the function of the corresponding orthologous gene in kiwifruit can be deduced from *Arabidopsis thaliana*. In addition, the spatial–temporal expression pattern of specific stress-responsive genes is an important part of plant stress response [34]. Therefore, the *AcMYBs*, which is in the same subgroup as the *AtMYBs* reported in phylogenetic analysis and which changes significantly in stress-treated transcriptional profiles, is comprehensively analyzed to screen *R2R3-MYB* TFs in kiwifruit that respond to stress. The results show that Achn121951, which was clustered into one branch with *AtMYB60* (AT1G08810.1) in the S1 subgroup, was significantly up-regulated under high-temperature stress; Achn173251, which was clustered into one branch with *AtMYB102* (AT4G21440.1) in the S11 subgroup, significantly increased under drought stress; Achn215991, which was clustered into one branch with *AtMYB61* (AT1G09540.1) in the S13 subgroup, was significantly down-regulated under drought stress; Achn324811, which was clustered into one branch with *AtMYB2* (AT2G47190.1) in subgroup S20, was significantly down-regulated under drought stress. All of them might be involved in the plant stress response.

Relevant studies have shown that *AtMYB102* [18] is involved in trauma and osmotic stress responses. *AtMYB60* is only expressed in guard cells, which is necessary for photoinduced stomatal opening. At the same time, the pore size of its mutant is reduced, helping to limit water loss during drought [16]. As for *AtMYB61*, it can coordinate a small network of downstream target genes required for several aspects of plant growth and development, such as xylem formation and xylem cell differentiation, as well as lateral root formation, and act as a transcriptional regulator of stomatal closure [35]. *AtMYB2* is involved in the regulation of ABA-induced genes under drought stress, and at the same time, it forms a complex with all known glucosinolate-related *MYBs* to regulate the biosynthesis of glucosinolate [19]. Notably, the responses of *AtMYB61* and *AtMYB2* to stress involved the regulation of phenylpropanoid pathway. Studies have shown that the response of *R2R3-MYB* gene to stress is often accompanied by changes in the content of phenylpropanoid pathway, such as flavonoid, anthocyanin and lignin [4,5,6]. Moreover, their distribution of metabolic flux is one of the means for plants to cope with changes in the external environment [6]. In particular, the lignin biosynthesis pathway [7], which is a biosynthesis center, is more significantly affected. Therefore, it is speculated that it may affect lignin biosynthesis and thus respond to stress.

**Figure 6:**
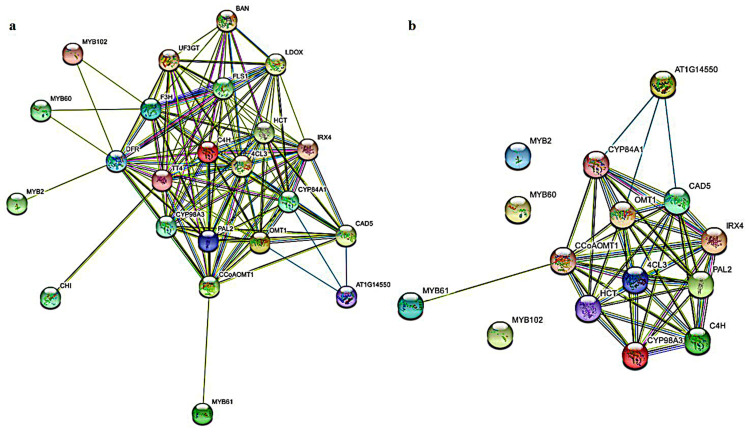
Protein regulatory network of *AcMYBs* protein in kiwifruit. (**a**) Protein regulatory networks of *MYBs* and structural genes of phenylpropanoid pathway. (**b**) Protein regulatory networks of *MYBs* and structural genes of lignin metabolic pathway. The purple line represents that interaction of the experimental results. Yellow-green, black and blue lines represent text mining, co-expression and protein homology, respectively.

The String website was used to predict the functional relationship between MYBs proteins of MYB2, MYB60, MYB61 and MYB102 in kiwifruit and related genes from the phenylpropanoid pathway (*Arabidopsis thaliana* as the species model). The connected genes in this network may have close functional ties. *MYB60*, *MYB2* and *MYB102* could be indirectly associated with caffeoyl-CoA O-methyltransferase (*CCoAOMT*), cinnamate 4-hydroxylase (*C4H*), ferulate-5-hydroxylase (*F5H*), 4-coumarate-CoA ligase (*4CL*), caffeic acid O-methyltransferase (*COMT*), phenylalanine ammonia-lyase (*PAL*), coumarate 3-hydroxylase (*C3′H*), cinnamyl alcohol dehydrogenase (*CAD*), shikimate O-hydroxycinnamoyltransferase (*HCT*), p-coumaryl alcohol by cinnamoyl-CoA reductase (*CCR*) and peroxidase (*POD*) through flavanone 3-hydroxylase (*F3H*), dihydro-flavonol 4-reductase (*DFR*), flavonol synthase (*FLS*), leucoanthocyanidin dioxygenase (*LDOX*), UDP flavonoid glucosyl transferase (*UFGT*) and other anthocyanin pathway-related genes (Figure 6a). There were direct functional relations or interaction relations between *MYB61* and *CCoAOMT*, *C4H*, *F5H*, *4CL*, *COMT*, *PAL*, *C3′H*, *CAD*, *HCT*, *CCR* and *POD* (Figure 6b). Therefore, it was speculated that the screened *MYB2*, *MYB60*, *MYB61* and *MYB102* might be related to a certain function of lignin biosynthesis. It was suggested that the lignin biosynthesis genes might be regulated by *MYBs*.

### 2.7. The Analysis of the MYB-Binding Site of the Candidate Genes from the Phenylpropanoid Pathway

**Figure 7:**
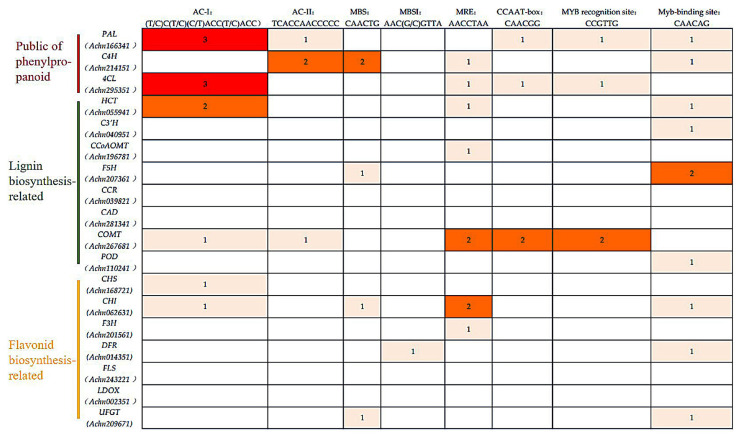
*MYB-binding* site of the candidate genes from the phenylpropanoid pathway.

Studies of *MYB-binding* sites have shown that cis elements that are generally rich in adenine and cytosine residues can be recognized by *MYB* TFs, such as *MYB-binding* site ((T/C) AAC (G/T) G (A/C/T) (A/C/T)), MBSI ((C/T) NGTT (A/G)), AC elements (ACC (A/T) A (A/C) (T/C) and ACC (A/T) (A/C/T) (A/C/T)) [36,37]. To identify whether the candidate genes from the phenylpropanoid pathway are regulated by the *AcMYBs*, the cis-acting elements of the 2 kb promoter upstream of the start codon of related genes were analyzed, and genes with the *MYB-binding* sites were identified (Figure 7). The results showed that most flavonoid and lignin-biosynthesis-related genes in kiwifruit have *MYB-binding* sites, such as *MYB-binding* site, AC- element and so on, except for *CCR*, *CAD*, *FLS* and *LDOX*.

It was remarkable that ‘ACC’ was the core recognition motif in the AC elements [38], which has been found to be closely related to the benzene–propane metabolic pathway. Studies showed that *SlMYB75* [39] could bind to the ‘ACCTACCC’ motif of the target gene promoter and effectively induce anthocyanin accumulation in tomato tissues. Meanwhile, both *PtMYB4* and *EgMYB2* can bind to the AC elements to regulate lignin biosynthesis [37]. Moreover, BplMYB46 protein [40] can also directly bind to the ‘ACCACCT’ motif of downstream target gene promoter to promote lignin deposition and secondary cell wall biosynthesis. In this study, the promoter regions of lignin-biosynthesis-related genes in kiwifruit were rich in AC elements, suggesting that these genes might be regulated by *MYB* TFs and involved in lignin biosynthesis.

### 2.8. Expression Profiles of AcMYBs and Lignin-Biosynthesis-Related Genes in Different Tissues of Kiwifruit

**Figure 8:**
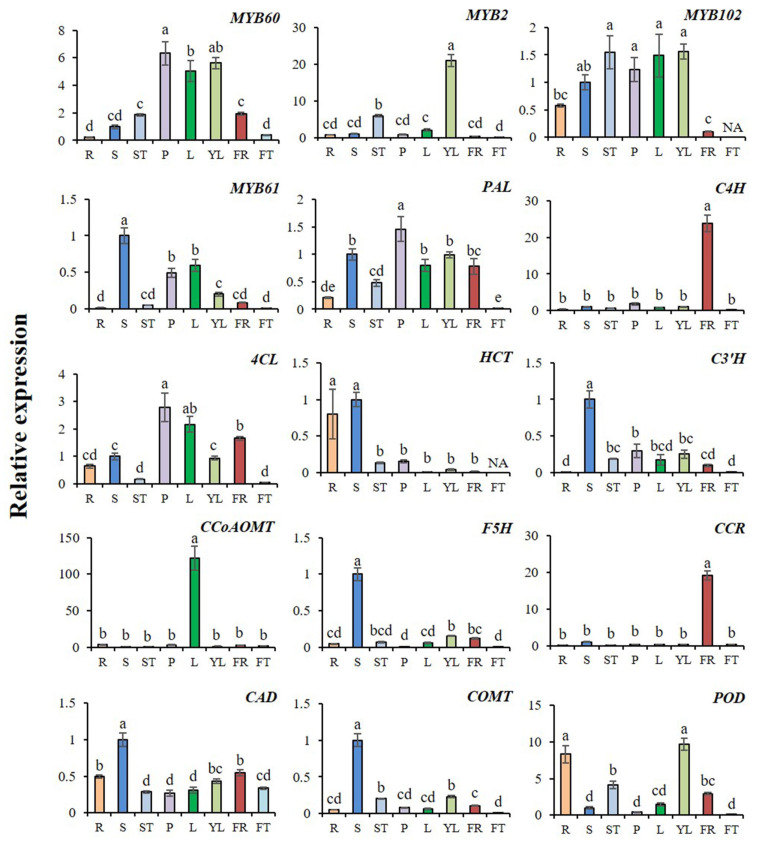
Relative expression levels of *AcMYBs* and lignin-biosynthesis-related genes in different tissues of kiwifruit. The expression level of this gene in the stem was taken as 1. The relative expression level of genes was calculated by normalization method. R. Root, S. stem, ST. stem tip, P. petiole, L. leaf, YL. young leaf, FR. flower, FT. fruit. Different superscript letters indicate significant differences between different treatments at *p* < 0.05 level. NA indicates that the expression level is lower than the minimum value detectable by the instrument.

The expression profiles of *AcMYBs* and lignin-biosynthesis-related genes in different tissues of kiwifruit were analyzed (Figure 8). The results showed that the screened *MYBs* generally had high expression in stem or stem tip, petiole, young leaf or leaf, but low expression in fruit. As for the related genes from lignin biosynthesis, except for the high expression of *AcC4H* and *AcCCR* in flowers, the rest were generally expressed in stems or stem tips, petioles, leaves or young leaves, and it was noted that *AcHCT*, *AcCAD* and *AcPOD* also had high expression in roots.

Correlation analysis showed (Table 2) that *AcMYB102* and *AcMYB61* had a very significant positive correlation with the initial response enzyme gene *AcPAL* of the common biosynthesis pathway of phenylpropane. *AcMYB60* was closely related to the initial response enzyme gene *AcPAL* and the ligase gene *Ac4CL*. The expression levels of related genes, such as lignin-related hydroxylases *AcC3′H* and *AcF5H*, reductase gene *AcCAD* and methylase gene *AcCOMT*, also showed a significant positive correlation with *AcMYB61*. It should be noted that *AcMYB2* was closely related to lignin biosynthesis and degradation, as well as to the related gene of suberized *AcPOD*. Based on these results, it could be speculated that there was co-expression between *AcMYB60*, *AcMYB2*, *AcMYB102* and *AcMYB61* or lignin-biosynthesis-related genes during plant growth and development.

### 2.9. Determination of Lignin Autofluorescence in Different Tissues of Kiwifruit

**Figure 9:**
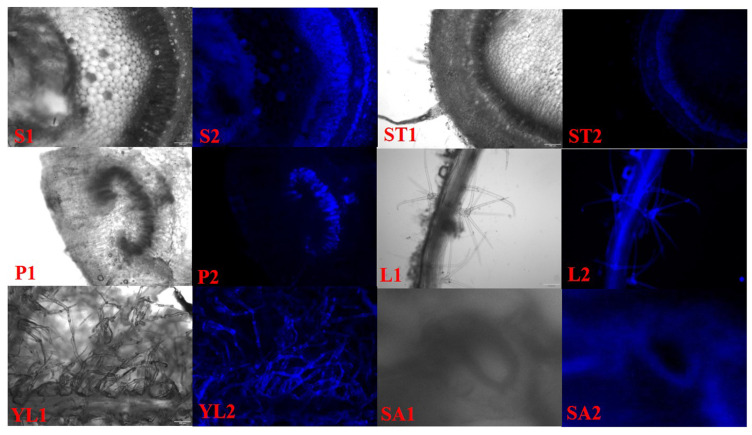
Lignin sections in different tissues of kiwifruit. S. stem, ST. stem tip, P. petiole, L. leaf, YL. young leaf, SA. stomata; 1. bright field, 2. ultraviolet (UV).

Based on the autofluorescence characteristics of lignin under ultraviolet light, it was found that the deposition of lignin in different tissues of kiwifruit was different (Figure 9). It was mainly deposited in the phloem and conducting tissues of the stem, and it was also deposited in the veins, villi and inner wall of guard cells of the leaves. Secondly, a small amount of deposition was also observed in the phloem of stem tip and the conducting tissue of petiole. This result was consistent with the expression profiles of *AcMYBs* and lignin-biosynthesis-related genes in different tissues. Therefore, it was speculated that the screened *AcMYB2*, *AcMYB60*, *AcMYB61* and *AcMYB102* might be related to the existence of a certain function of lignin deposition.

### 2.10. Expression Profiles of AcMYBs and Lignin-Biosynthesis-Related Genes in Kiwifruit under Different Stresses

**Figure 10:**
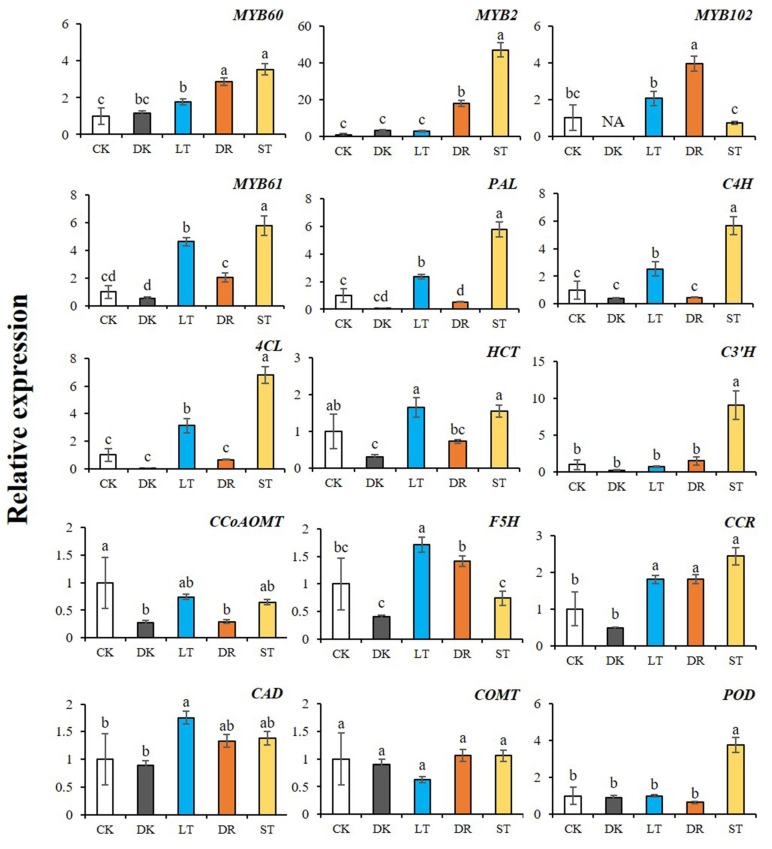
Relative expression levels of *AcMYBs* and lignin-biosynthesis-related genes in kiwifruit under different stresses. CK. control, DK. dark, LT. low temperature, DR. PEG simulation of drought, ST. salt. Different superscript letters indicate significant differences between different treatments at *p* < 0.05 level. NA indicates that the expression level is lower than the minimum value detectable by the instrument.

Through cis-acting element analysis, it was found that the four screened *AcMYBs* members mostly contained stress-responsive elements, such as drought, low temperature and hypoxia, as well as light-responsive elements (Table S10). Therefore, their expression levels were determined by qRT-PCR under different abiotic stresses (dark, low temperature, PEG simulation of drought and salt) (Figure 10). From the results, we could find that both *AcMYB2* and *AcMYB102* could respond to salt stress, while *AcMYB60* and *AcMYB61* responded to low temperature and salt stress. As for the relative expression levels of lignin-biosynthesis-related genes, they were increased under low-temperature stress and salt stress. In addition, under dark conditions, *AcMYBs* and lignin-biosynthesis-related genes were mostly expressed at a relatively low level, suggesting that dark conditions might be unfavorable for lignin accumulation. Therefore, it was speculated that the four selected *AcMYBs* might affect lignin biosynthesis and respond to stress.

Correlation analysis (Table 3) showed that *AcMYB60*, *AcMYB2* and *AcMYB61* had highly significant positive correlations with two initial response enzyme genes and one ligase gene *AcPAL*, *AcC4H* and *Ac4CL* of the common biosynthesis pathway of phenylpropane, respectively. Among the related genes, including lignin-related hydroxylases *AcF5H*, *AcC3′H* and methylase genes *AcCOMT*, *AcCCoAOMT* and reductase genes *AcCCR* and *AcCAD*, *AcMYB61* was closely related to these genes, except for the methylase genes *AcCCoAOMT* and *AcCOMT*. As for *AcMYB60* and *AcMYB2*, they were closely related to the *AcC3′H* and reductase gene *AcCCR* that determine the biosynthesis of G-and S-type lignin, as well as to the biosynthesis and degradation of lignin, and the related gene of *AcPOD* that is responsible for cork-rization. *AcMYB102* was closely related to hydroxylase F5H. Based on these results, it could be speculated that *AcMYB60*, *AcMYB2*, *AcMYB102* and *AcMYB61* might co-express with lignin-biosynthesis-related genes in the process of plant stress response.

## 3. Discussion

In recent years, a variety of plants have systematically identified the *MYB* TF family through genomic analysis [41,42]. However, no literature has been reported regarding the responses of members of the *MYB* family in kiwifruit to stress and their responses to lignin-biosynthesis-related genes. In this study, 181 *AcMYB* TFs were identified in the kiwifruit genome and subjected to chromosome distribution analysis, gene duplication event analysis, conservative domain identification, phylogenetic analysis, gene structure and protein motif analysis. Furthermore, *AcMYBs* participating in stress resistance was screened out based on transcriptomic data. Protein regulatory network prediction and promoter cis-acting element analysis were conducted to explore the responses of selected *AcMYBs* genes to lignin-biosynthesis-related genes and stress responses.

The results showed that the identified *AcMYBs* gene in kiwifruit was different in amino acid sequence length, physical and chemical properties (such as molecular weight and isoelectric point) and gene structure. It reflected the complexity and functional diversity of kiwifruit *MYBs* gene to a certain extent. At the same time, kiwifruit *MYBs* gene has tandem repeat events and has a closer collinearity with *Arabidopsis thaliana*. Notably, in this identification, we referred to the reported functions of members of *AtMYBs* in *Arabidopsis thaliana* and transcriptomic data of the previous research group, and we identified four *MYBs* that might participate in stress response. According to the functions, it was speculated that they might participate in the phenylpropanoid pathway, in which the lignin biosynthesis pathway was the central pathway [7]. It was suggested that it might affect lignin biosynthesis to a certain extent. Therefore, the prediction of protein regulatory network was performed for its interaction with lignin-biosynthesis-related genes. It was found that *AcMYB61* could be directly associated with lignin-biosynthesis-related genes, while *AcMYB2*, *AcMYB60* and *AcMYB102* could indirectly affect lignin metabolism through anthocyanin-biosynthesis-related genes. At the same time, except for *AcCCR* and *AcCAD*, the promoter regions of other lignin-biosynthesis-related genes contained *MYB-binding* sites, such as AC-element, MRE, MBS and so on (Figure 7). Therefore, it was speculated that lignin-biosynthesis-related genes might be regulated by *MYBs*.

It should be noted that the selected *AcMYB2*, *AcMYB60*, *AcMYB61* and *AcMYB102* mostly expressed in stems or stem tips, petioles, leaves or young leaves with high expression levels, and the expression profiles of lignin-biosynthesis-related genes showed a similar trend. The results of correlation analysis also indicated that these four genes might co-express with lignin-biosynthesis-related genes in the process of plant growth and development. Moreover, the results of lignin autofluorescence measurement showed that lignin did deposit in guard cells, vein and villous parts of kiwifruit leaves, phloem and translocation tissue of stem, translocation tissue of petiole and phloem of stem tip.

In addition, the selected *AcMYBs* also showed responses to low-temperature stress, salt stress or drought stress, which were consistent with the results of their cis-acting element analysis. At the same time, the expression levels of structural genes for lignin metabolism were increased under low temperature or osmotic stress. The results of correlation analysis showed that the expression changes of *AcMYB60*, *AcMYB2* and *AcMYB61* under stress had a significant positive correlation with the lignin-biosynthesis-related genes. It indicated that there might be co-expression effect therebetween, so as to resist stress. Based on this speculation, the up-regulation of kiwifruit *MYB61* expression under low temperature or osmotic stress caused the up-regulation of lignin-biosynthesis-related genes, such as *AcPAL*, *AcC4H*, *Ac4CL*, *AcCCR*, *AcCAD* and *AcPOD*, which caused more lignin deposition and helped to resist external stress. This conclusion coincides with the research conclusion that the degree of lignification of soybean root system and wheat under salt stress is relatively high [43,44]. However, *AcMYB60*, *AcMYB2* and *AcMYB102* could indirectly up-regulate the expression of *AcCCR* or *AcCAD* and then deposit lignin to help resist stress.

## 4. Materials and Methods

### 4.1. Plant Materials and Abiotic Stress Treatments

The kiwifruit tissue, including stem tip, young leaf, root, stem, leaf, flower and fruit (*n* = 3), were collected from ‘Jinyan’ at a kiwifruit orchard in Pujiang County, Chengdu, China (29°28′ N, 119°53′ E). Samples were frozen in liquid nitrogen and stored at −80 °C using the RNA extract.

The sterile tissue culture seedlings of ‘Jinyan’ kiwifruit with consistent growth were selected for stress treatment. The 60 aseptic seedlings were equally divided into five groups for treatment, including control treatment group, dark treatment group, low temperature treatment group, PEG simulation of drought treatment group and salt treatment group. Except for the LT treatment group, which grew at 4 ± 2 °C, the other four treatment groups were all grown in the culture room at light/dark 16 h/8 h at 25 ± 2 °C. The DK treatment group was shaded with black cloth; the DR treatment group was treated with 40% (*w*/*v*) PEG-6000 under simulated drought stress; and the ST treatment group was simulated with 100 mmol/L NaCl. Three strains were repeated four times. Samples were taken at 48 h of stress and stored at −80 °C immediately after freezing in liquid nitrogen.

### 4.2. Identification and Physicochemical Properties Analysis of AcMYB TFs

The kiwifruit genome database from Kiwifruit Genome Database (http://kiwifruitgenome.org/ (accessed on 20 October 2021)) was compared and integrated with the transcriptomic data of kiwifruit under stresses previously sequenced by the research team. Predictions were made using the Plant TFDB database (http://plantfdb.Gao-lab.org/ (accessed on 21 October 2021)). *AtMYB* in *Arabidopsis thaliana* dataset (downloaded by TAIR (https://www.arabidopsis.org/ (accessed on 21 October 2021)) as a probe) bidirectional blast was performed using TBtools and the NCBI conservative domain database. Then, the hidden Markov model file of *MYB* gene family (PF00249) was downloaded from PFAM (http://pfam.xfam.org/ (accessed on 21 October 2021)) website and retrieved using hmmer 3.0. Combined with the above three methods, the search results were integrated to finally determine the *AcMYB-related* TFs and *AcMYB* TFs. The physical and chemical properties analyses of AcMYB protein in kiwifruit were performed using the online tool ProtParam (http://web.expasy.org/protparam/ (accessed on 25 October 2021)).

### 4.3. Chromosomal Distribution, Gene Duplication Events and Collinearity Analysis of AcMYBs

The chromosomal distribution of *AcMYB* gene was retrieved from the Kiwifruit Genome Database gff3 file and visualized by Map Inspect. Gene duplication events in the *AcMYB* TFs were detected using MC Scan X with default parameters. Additionally, the homology analysis atlas of kiwifruit, *Arabidopsis thaliana* and rice was constructed by using TBtools software.

### 4.4. Conservative Motif, Gene Structure Analysis and Phylogenetic Analysis of AcMYBs

The conserved motifs of *AcMYBs* were revealed using MEME (https://meme-suite.org/ (accessed on 21 October 2021)), and the gene structure was extracted and visualized using TBtools. For phylogenetic analysis, the sequences of AtMYBs protein in *Arabidopsis thaliana* downloaded by TAIR were used as reference, and the conservative domains of MYB protein in kiwifruit and *Arabidopsis thaliana* were preserved. After Clustal W alignment, a neighbor-joining (NJ) phylogenetic tree was constructed using Mega X, and the bootstrap value was set to 1000 repetitions.

### 4.5. Expression Analysis Based on Transcriptomic Data

Based on the transcriptomic data of kiwifruit under drought and high-temperature stresses sequenced by our research group at the early stage, the low-quality reading (padj > 0.05) was removed, and the transcriptional abundance of the gene was expressed as FPKM (number of fragments per million kilobases). log_2_(FPKM) was used for hierarchical clustering, and the results were visualized by TBtools.

### 4.6. Prediction of AcMYBs Protein Regulatory Network

The String website (http://string-db.org/ (accessed on 21 November 2021)) was used to analyze the protein regulatory network of AcMYBs protein in kiwifruit, with the model plant *Arabidopsis thaliana* as the species parameter.

### 4.7. MYB-Binding Site Analysis

The genomic DNA sequences of 2 kb upstream of the initiation codon of phenylpropanoid pathway-related candidate gene was extracted using TBtools software and submitted to PlantCARE database (http://bioinformatics.psb.ugent.be/webtools/plantcare/html/ (accessed on 22 November 2021)) for the cis-acting element and functional sites analysis in the promoter regions.

### 4.8. Extraction of Total RNA and qRT-PCR Analysis

The extraction of total RNA in kiwifruit referred to the modified CTAB-LiCl method [45]. The cDNA prepared by PrimeScriptTM RT reagent Kit with gDNA Eraser (TaKaRa, Dalian, China) was used as the template. Quantitative PCR was performed using TB Green^®^ Premix Ex TaqTM II (TaKaRa, Dalian, China). Gene-specific primers were designed using Primer 5.0 (Table S1). The 2^−ΔΔCQ^ method was used to quantify the relative expression level of the gene. *AcActin* (GenBank Accession No.EF063572) gene was used as the endogenous internal reference gene. Three biological and technical repetitions were completed for each sample based on qRT-PCR.

### 4.9. Determination of Lignin Autofluorescence in Different Tissues of Kiwifruit

Different tissues, such as leaves, petioles and stems of ‘Jinyan’ kiwifruit seedlings, were sectioned by hand, and the blue fluorescence of lignified tissues under ultraviolet (UV) fluorescence was observed under the fluorescence phase contrast micro-imaging system, according to the methods described by Jia [46] and Han [47].

### 4.10. Data Analysis

The experimental data were all expressed as mean standard error (M ± SE). The statistical analysis and chart drawing were performed using Excel 2010 software, while the significance analysis was performed using SPSS 20.0 software using one-way ANOVA or Tukey test (*p* < 0.05). The correlation analysis was a two-variable linear correlation analysis, expressed as Pearson correlation coefficient (R).

## Figures and Tables

**Table 1:** Classification and physicochemical properties of identified *AcMYB* TFs in kiwifruit.

Gene Type	Number	No. of aa	Protein Isoelectric	Molecular Weight (kDa)	Grand Average of Hydropathicity
*MYB-relative*	87	67–998	4.13–10.28	7.46–112.62	−1.232–−0.168
*R2R3-MYB*	91	117–1867	4.9–10.01	13.37–204.9	−0.996–−0.013
*3R-MYB*	3	488–1944	5.28–8.91	54.89–213.04	−0.341–−0.754
Total	181	67–1944	4.13–10.28	7.46–213.04	−1.232–−0.013

**Table 2:** Correlation between expression level of *AcMYBs* and lignin-biosynthesis-related genes in different tissues.

	*AcMYB60*	*AcMYB2*	*AcMYB102*	*AcMYB61*
*AcPAL*	0.776 **	0.212	0.540 **	0.626 **
*AcC4H*	−0.090	−0.195	−0.473 *	−0.212
*Ac4CL*	0.738 **	−0.166	0.282	0.475 *
*AcHCT*	−0.457 *	−0.250	−0.032	0.437 *
*AcC3′H*	0.032	0.002	0.317	0.874 **
*AcCCoAOMT*	0.370	−0.121	0.344	0.325
*AcF5H*	−0.253	−0.054	0.085	0.760 **
*AcCCR*	−0.145	−0.210	−0.510 *	−0.214
*AcCAD*	−0.401	−0.115	−0.149	0.585 **
*AcCOMT*	−0.196	0.024	0.192	0.752 **
*AcPOD*	0.041	0.696 **	0.248	−0.420 *

The data in the table are value of Pearson correlation coefficient (R value, *n* = 9), * indicates significant correlation at *p* < 0.05 level. ** indicates significant correlation at *p* < 0.01 level.

**Table 3:** Correlation between expression level of *AcMYBs* and lignin-biosynthesis-related genes under different stresses.

	*AcMYB60*	*AcMYB2*	*AcMYB102*	*AcMYB61*
*AcPAL*	0.702 **	0.830 **	−0.180	0.903 **
*AcC4H*	0.679 **	0.802 **	−0.203	0.907 **
*Ac4CL*	0.703 **	0.810 **	−0.148	0.931 **
*AcHCT*	0.460	0.383	0.158	0.862 **
*AcC3′H*	0.799 **	0.952 **	−0.158	0.738 **
*AcCCoAOMT*	−0.113	−0.093	−0.081	0.240
*AcF5H*	0.194	−0.179	0.782 **	0.349
*AcCCR*	0.892 **	0.756 **	0.451	0.888 **
*AcCAD*	0.507	0.222	0.525 *	0.741 **
*AcCOMT*	0.430	0.378	0.207	−0.030
*AcPOD*	0.691 **	0.885 **	−0.320	0.739 **

The data in the table are value of Pearson correlation coefficient (R value, *n* = 9), * indicates significant correlation at *p* < 0.05 level. ** indicates significant correlation at *p* < 0.01 level.

## Data Availability

All data are reported in this manuscript.

## Supplementary Materials

The following supporting information can be downloaded at: https://www.mdpi.com/article/10.3390/ijms23042323/s1.
