# Supplementary material for: Genome-Wide Identification of MYB Transcription Factors and Screening of Members Involved in Stress Response in Actinidia
Source: Int J Mol Sci. 2022 Feb 19;23(4):2323. doi: 10.3390/ijms23042323 (PMC8875009; doi:10.3390/ijms23042323)
Supplement: Supplementary file 1 [file ijms-23-02323-s001.zip › ijms-1556499-SI.pdf]

Table S1. Primer sequence for gene expression analysis.

Primer Name	Primer Sequense (5'-3')
qAcActin-S	GCAGGAATCCATGAGACTACC
qAcActin-A	GTCTGCGATACCAGGGAACAT
qAcMYB61-S	CCCCAACACCCACAAACCG
qAcMYB61-A	TTCTCCGAATCGCCACCCT
qAcActin-S	GCAGGAATCCATGAGACTACC
qAcActin-A	GTCTGCGATACCAGGGAACAT
qAcPAL-S	CAATGGCTCGGCCCACAGA
qAcPAL-A	CGATTGGCGTGCCCTGAAAA
qAcC4H-S	GCCCGACACCTACAAACTGCCCTAC
qAcC4H-A	GCGAGCCACCAAGCGTTTACCA
qAc4CL-S	CTTCTTGTGAGCCACCCGTCCAT
qAc4CL-A	GACTGCTGCTTCTGTAAGTTCAAACCC
qAcC3'H-S	CAAGAAGAGTTGGACCGTGTAATTGGG
qAcC3'H-A	AAGCATCAGCGGCGTGGGAG
qAcCCoAOMT-S	GTAATAGCGATTGACCAAAGCAGAG
qAcCCoAOMT-A	TGGCGTCGGATTGAATGAAG
qAcF5H-S	AGAAACAGTGGCGTCGGCGATAGA
qAcF5H-A	TCTCCTTGAGGGCGCACTTGAGATA
qAcCCR-S	GAAGAACTACACCAACTCGGTCCAGG
qAcCCR-A	CACGCTTTCGGCGCAAAGATA
qAcCAD-S	CAGGGAGCTTCATAGGGAGCATG
qAcCAD-A	CGCTCGAAGGCGGTGTTGAT
qAcCOMT-S	ATGTTGGGGGTGACATGTTT
qAcCOMT-A	AACGCGTCGTAGCAGTTTTT
qAcPOD-S	GCTTGCCCTGCTAGTGGGAATA
qAcPOD-A	TGAAACTGGAAGGCTTTGTGCT

Table S2. Physicochemical properties of *AcMYB-relative* TFs in kiwifruit.

Gene ID	Molecular weight (kD)	Number of amino acid	Protein isoelectric	Grand average of hydropathicity
Achn216541	38.28	350	6.4	−0.592
Achn049881	38.73	351	6.84	−0.494
Achn062481	26.28	237	9.42	−0.701
Achn319301	34.04	309	8.7	−0.572
Achn100041	36.58	338	8.5	−0.473
Achn001361	36.70	340	6.59	−0.591
Achn141911	39.16	361	6.31	−0.559
Achn121851	24.60	219	8.81	−0.502
Achn183551	31.69	292	8.37	−0.582
Achn217431	38.80	353	9.64	−0.392
Achn137671	36.98	330	9.38	−0.435
Achn356431	33.09	301	9.25	−0.495

Achn009551	30.84	305	8.99	−0.692
Achn036201	33.49	331	8.99	−0.792
Achn141271	30.46	269	8.27	−0.575
Achn145641	32.85	303	6.88	−0.611
Achn238851	31.52	278	8.98	−0.838
Achn268811	18.27	173	10.27	−0.397
Achn296511	33.67	302	10.28	−0.839
Achn188021	29.79	270	9.18	−0.435
Achn351781	22.89	202	9.01	−0.673
Achn093361	50.61	463	5.57	−0.724
Achn191781	50.61	463	5.57	−0.724
Achn131251	10.65	94	9.13	−0.891
Achn197601	10.93	94	6.73	−0.889
Achn232671	98.11	88	6.72	−0.887
Achn255701	24.89	226	5.62	−0.62
Achn258201	45.54	423	5.84	−0.544
Achn268801	21.50	197	5.2	−1.132
Achn357831	11.76	105	9.75	−0.75
Achn372411	11.33	100	9.45	−0.888
Achn373061	13.53	120	9.3	−0.749
Achn232661	9.79	88	6.73	−0.925
Achn006751	23.64	209	9.25	−0.552
Achn147111	46.99	423	9.2	−0.461
Achn198661	100.73	907	6.32	−0.615
Achn115241	106.28	959	7.29	−0.576
Achn214091	66.92	590	8.95	−0.587
Achn115281	33.12	302	9.43	−0.748
Achn104781	60.99	539	8.69	−0.468
Achn034581	33.82	310	8.58	−0.387
Achn198701	21.92	202	5.29	−0.619
Achn363591	31.93	294	8.63	−0.453
Achn168281	17.85	162	8.87	−0.846
Achn090351	43.24	372	6.77	−0.946
Achn154591	33.81	298	8.77	−0.808
Achn033551	31.27	288	6.67	−0.535
Achn365361	24.23	212	6.07	−0.81
Achn029591	24.23	212	6.07	−0.81
Achn218481	32.87	286	9.22	−1.017
Achn207851	22.33	200	5.97	−0.62
Achn371351	31.02	279	8.46	−0.667
Achn136031	25.26	221	6.36	−0.892
Achn104391	25.57	223	6.55	−1.042
Achn307721	21.23	189	4.9	−0.646
Achn335391	60.03	536	4.45	−0.747

Achn138221	48.03	425	4.56	−0.68
Achn076861	112.63	998	5.57	−0.842
Achn302321	29.36	263	8.52	−0.591
Achn018291	29.66	268	5.97	−0.569
Achn052151	33.11	294	4.97	−0.422
Achn095271	55.81	495	7.01	−0.47
Achn138771	13.80	123	9.25	−0.53
Achn165731	30.49	271	4.83	−0.859
Achn167511	7.46	67	7.71	−0.337
Achn172911	23.56	203	4.78	−0.913
Achn205651	10.82	95	4.62	−0.638
Achn212251	30.84	273	8.99	−0.692
Achn212761	29.08	258	6.59	−0.889
Achn229041	29.89	267	5.06	−0.578
Achn242001	13.85	121	10.01	−0.542
Achn246461	22.38	196	4.78	−0.606
Achn248041	28.88	258	4.72	−0.601
Achn264671	31.88	283	6.17	−0.689
Achn270991	24.56	221	7.64	−0.653
Achn284031	34.31	303	4.92	−0.8
Achn289751	19.59	176	8.19	−0.713
Achn297341	15.19	131	7.83	−0.866
Achn311101	8.59	77	4.96	−0.168
Achn315561	21.42	188	5.96	−1.039
Achn318681	32.90	295	6.3	−0.481
Achn334541	24.40	221	4.82	−0.375
Achn338441	34.89	306	6.03	−0.765
Achn340541	18.98	171	8.48	−0.687
Achn346721	29.30	264	5.41	−0.559
Achn347951	21.58	192	9.65	−0.77
Achn374211	14.05	124	4.13	−0.494
Achn378631	29.16	260	5.04	−0.574
Achn389751	25.84	222	6.42	−0.972

Note: Achn093361/Achn191781 and Achn365361/Achn029591 were redundant genes with identical sequences. As the chromosomal distribution of Achn093361 and Achn365361 was unclear, Achn191781 and Achn029591 were reserved for subsequent analysis.

Table S3. Physicochemical properties of *AcR2R3-MYB* TFs in kiwifruit.

Gene ID	Molecular weight (kD)	Number of amino acid	Protein isoelectric	Grand average of hydropathicity
Achn001581	28.30	250	5.93	−0.668
Achn005711	31.33	275	5.04	−0.691
Achn007681	49.92	458	6.36	−0.604
Achn012091	54.54	479	9.34	−0.559

Achn013291	46.48	431	6.38	−0.631
Achn013391	23.28	207	9.32	−0.735
Achn020361	27.00	241	8.97	−0.72
Achn020551	33.38	297	7.18	−0.753
Achn021211	45.66	398	5.67	−0.806
Achn027081	31.41	277	8.66	−0.595
Achn030851	28.37	248	5.15	−0.491
Achn031311	36.03	318	5.54	−0.526
Achn035581	57.76	510	6.3	−0.571
Achn040631	41.17	358	9.57	−0.944
Achn047241	31.57	282	6.76	−0.58
Achn051601	44.16	389	4.99	−0.805
Achn055151	44.86	409	5.87	−0.479
Achn064821	28.12	247	6.44	−0.564
Achn077281	48.73	429	5.31	−0.758
Achn081171	30.54	267	5.82	−0.802
Achn081181	32.66	290	5.85	−0.629
Achn089571	28.35	250	5.32	−0.626
Achn092691	29.29	258	5.52	−0.639
Achn098041	49.57	443	8.13	−0.719
Achn102731	36.88	320	5.11	−0.774
Achn108411	34.60	299	8.85	−0.841
Achn109311	47.90	428	6.89	−0.996
Achn109411	41.19	360	9.5	−0.921
Achn111201	110.38	985	7.79	−0.013
Achn111951	28.66	249	6.01	−0.565
Achn116791	24.84	215	9.45	−0.664
Achn121951	31.94	290	6.23	−0.645
Achn132371	31.40	275	5.22	−0.608
Achn142371	49.97	441	6.86	−0.767
Achn143561	25.97	228	9.12	−0.784
Achn144741	37.07	329	8.57	−0.643
Achn148821	32.19	287	8.77	−0.846
Achn169441	27.39	242	6.61	−0.804
Achn171911	39.39	344	5.92	−0.972
Achn172271	33.87	290	5.96	−0.913
Achn172901	27.34	240	8.1	−0.672
Achn173251	36.27	322	5.4	−0.541
Achn182891	29.98	267	5.94	−0.833
Achn195411	33.86	295	6.05	−0.858
Achn195891	22.90	197	8.81	−0.978
Achn197141	32.73	292	8.58	−0.647
Achn198731	35.09	311	6.85	−0.429
Achn199471	30.76	272	8.4	−0.74

Achn202121	44.34	390	5.44	−0.819
Achn204961	35.53	315	6.42	−0.637
Achn211821	43.98	401	5.74	−0.369
Achn215991	46.63	418	7.69	−0.8
Achn222811	37.31	330	6.05	−0.528
Achn223911	61.10	562	5.22	−0.628
Achn225741	110.77	1010	5.09	−0.582
Achn228371	31.86	276	6.32	−0.74
Achn229071	43.27	399	6.24	−0.683
Achn234881	30.75	275	6.55	−0.72
Achn242001	13.85	121	10.01	−0.542
Achn256021	34.95	309	9.6	−0.817
Achn267491	38.22	340	6.73	−0.599
Achn281601	19.86	177	6.51	−0.589
Achn284401	33.44	290	9.51	−0.942
Achn289001	29.85	255	6.81	−0.883
Achn294101	25.62	224	8.97	−0.769
Achn297221	24.89	217	6.36	−0.809
Achn298521	13.38	117	9.84	−0.469
Achn307151	41.46	381	5.79	−0.549
Achn307261	29.12	252	5.95	−0.752
Achn313331	35.73	320	6.31	−0.512
Achn315001	28.89	260	7.67	−0.483
Achn317571	28.70	254	5.22	−0.619
Achn322351	25.71	225	9.08	−0.794
Achn324811	31.45	274	5.59	−0.789
Achn337181	51.47	472	8.46	−0.692
Achn345001	43.41	382	5.57	−0.399
Achn352391	34.51	304	5.49	−0.663
Achn358581	46.35	403	6.54	−0.773
Achn358591	27.91	240	9.42	−0.41
Achn366791	33.25	296	8.95	−0.834
Achn368681	204.91	1867	5.14	−0.51
Achn370001	62.42	573	4.9	−0.582
Achn372141	30.60	277	8.76	−0.553
Achn375641	48.32	433	8.65	−0.597
Achn377521	33.06	295	8.79	−0.859
Achn380841	51.04	453	6.3	−0.825
Achn382461	27.14	235	8.93	−0.718
Achn382471	30.42	261	5.43	−0.876
Achn384891	44.43	407	6.33	−0.576
Achn389471	24.48	214	5.91	−0.836

Table S4. Physicochemical properties of *Ac3R-MYB* TFs in kiwifruit.

Gene ID	Molecular weight (kD)	Number of amino acid	Protein isoelectric	Grand average of hydropathicity
Achn295821	54.89	488	8.75	−0.663
Achn380251	66.06	592	8.91	−0.754
Achn361141	213.04	1944	5.28	−0.341

Table S5. Ka, Ks and Ka/Ks values of *AcMYB* collinearity pairs in kiwifruit.

Gene1	Gene2	Ka	Ks	Ka/Ks	Duplication Type
Achn232661	Achn232671	0.004903982	0.017022007	0.2880965	Tandem
Achn382471	Achn382461	0.385373806	1.593885254	0.2417827	Tandem
Achn001361	Achn100041	0.127831467	0.645064836	0.1981684	Segmental
Achn001361	Achn141911	0.038090917	0.215931526	0.1764028	Segmental
Achn009551	Achn141271	0.236844537	1.012030071	0.2340291	Segmental
Achn036201	Achn141271	0.335736878	2.174847674	0.1543726	Segmental
Achn036201	Achn154591	0.041624805	0.166539333	0.2499398	Segmental
Achn049881	Achn216541	0.030180637	0.117636522	0.2565584	Segmental
Achn062481	Achn319301	0.043217966	0.243831358	0.1772453	Segmental
Achn137671	Achn183551	0.397643749	1.813560741	0.2192613	Segmental
Achn121851	Achn137671	0.059727829	0.11514146	0.5187343	Segmental
Achn137671	Achn217431	0.23605886	0.69424229	0.3400237	Segmental
Achn141271	Achn154591	0.32903019	1.958400281	0.1680097	Segmental
Achn141271	Achn212251	0.236844537	1.012030071	0.2340291	Segmental
Achn183551	Achn217431	0.313756346	1.688831857	0.1857831	Segmental
Achn121851	Achn217431	0.153499241	0.611090915	0.2511889	Segmental
Achn131251	Achn372411	0.348118169	3.099372246	0.1123189	Segmental
Achn197601	Achn232661	0.264336334	2.385717443	0.1107995	Segmental
Achn232661	Achn373061	0.104827225	0.458838968	0.2284619	Segmental
Achn006751	Achn373061	0.243704694	0.416324931	0.5853714	Segmental
Achn006751	Achn232661	0.143377292	0.626219731	0.2289568	Segmental
Achn115241	Achn198661	0.085345765	0.169686	0.5029629	Segmental
Achn115281	Achn198701	0.044252465	0.175515384	0.2521287	Segmental
Achn034581	Achn363591	0.081339066	0.149212143	0.5451236	Segmental
Achn154591	Achn212251	0.33304233	2.070675054	0.1608376	Segmental
Achn297341	Achn389751	0.069499005	0.187079219	0.3714951	Segmental
Achn021211	Achn297341	0.476783864	1.536284212	0.3103487	Segmental
Achn289751	Achn340541	0.12253547	0.445018282	0.2753493	Segmental
Achn340541	Achn347951	0.266877753	0.744142591	0.3586379	Segmental
Achn020361	Achn340541	0.25248451	0.963011634	0.2621822	Segmental
Achn289751	Achn347951	0.240811549	1.169113777	0.2059779	Segmental
Achn315561	Achn338441	0.105231443	0.336497229	0.312726	Segmental
Achn020361	Achn347951	0.17595687	0.352827046	0.4987057	Segmental
Achn104391	Achn136031	0.040711516	0.264814086	0.1537362	Segmental
Achn202121	Achn389751	0.643977423	2.349754989	0.2740615	Segmental

Achn021211	Achn389751	0.676712041	1.923712018	0.3517741	Segmental
Achn248041	Achn378631	0.625282683	4.168199973	0.1500126	Segmental
Achn248041	Achn346721	0.218157723	0.627451928	0.3476883	Segmental
Achn165731	Achn248041	0.456420882	1.409156061	0.3238966	Segmental
Achn018291	Achn248041	0.221848851	0.751402681	0.2952463	Segmental
Achn165731	Achn378631	0.37548329	0.806430743	0.4656113	Segmental
Achn018291	Achn346721	0.051058617	0.216661837	0.2356604	Segmental
Achn229041	Achn234881	0.156328053	0.413282364	0.3782597	Segmental
Achn052151	Achn132371	0.322696324	0.86568036	0.3727661	Segmental
Achn313331	Achn318681	0.518647933	1.898787619	0.2731469	Segmental
Achn302321	Achn375641	0.218899901	0.443195482	0.4939128	Segmental
Achn215991	Achn242001	0.378206301	3.786543109	0.0998817	Segmental
Achn311101	Achn374211	0.327633592	0.492675921	0.6650083	Segmental
Achn138221	Achn335391	0.233254952	0.616808814	0.3781641	Segmental
Achn215991	Achn242001	0.378206301	3.786543109	0.0998817	Segmental
Achn297221	Achn389471	0.053927853	0.28759556	0.1875128	Segmental
Achn143561	Achn322351	0.170402865	0.778299789	0.2189425	Segmental
Achn081181	Achn358591	0.03612048	0.30239068	0.1194497	Segmental
Achn169441	Achn182891	0.111945701	0.278307742	0.4022371	Segmental
Achn030851	Achn111951	0.07630232	0.175877566	0.4338377	Segmental
Achn001581	Achn089571	0.056430838	0.377874441	0.1493375	Segmental
Achn001581	Achn317571	0.148000873	0.928468065	0.1594033	Segmental
Achn001581	Achn092691	0.156798661	0.951928354	0.1647169	Segmental
Achn089571	Achn317571	0.146370668	0.968708328	0.1510988	Segmental
Achn089571	Achn092691	0.134235261	0.937922061	0.1431198	Segmental
Achn092691	Achn317571	0.04895842	0.264740378	0.1849299	Segmental
Achn081171	Achn358581	0.081692483	0.198708361	0.4111175	Segmental
Achn005711	Achn132371	0.065170607	0.221072574	0.2947928	Segmental
Achn204961	Achn372141	0.15788043	0.254152739	0.6212029	Segmental
Achn047241	Achn267491	0.062015455	0.135034085	0.4592578	Segmental
Achn108411	Achn172271	0.146501453	0.710768451	0.206117	Segmental
Achn229071	Achn377521	0.054918623	0.285649462	0.1922588	Segmental
Achn031311	Achn345001	0.240018001	0.79604036	0.3015149	Segmental
Achn031311	Achn111201	0.078777145	0.227057844	0.3469475	Segmental
Achn267491	Achn358581	0.678862841	1.963618872	0.3457203	Segmental
Achn171911	Achn380841	0.234755195	0.963345041	0.2436876	Segmental
Achn040631	Achn109411	0.029768273	0.252751634	0.1177768	Segmental
Achn111201	Achn345001	0.35153059	1.041037716	0.3376733	Segmental
Achn021211	Achn202121	0.037243194	0.270297617	0.1377859	Segmental
Achn055151	Achn211821	0.091645619	0.209222003	0.4380305	Segmental
Achn007681	Achn211821	0.370801559	1.348827208	0.2749066	Segmental
Achn007681	Achn055151	0.372681937	1.349783409	0.276105	Segmental
Achn109311	Achn337181	0.299481455	0.58847474	0.5089113	Segmental
Achn163941	Achn337181	0.031034046	0.069707128	0.4452062	Segmental

Achn223911	Achn370001	0.041528968	0.096216335	0.4316208	Segmental

Table S6. *MYB* syntenic pairs of kiwifruit and *Arabidopsis thaliana.*

Kiwifruit	Arabidopsis
Achn049881	AT3G16350
Achn317571	AT4G12350
Achn109411	AT2G37630
Achn001581	AT4G12350
Achn001361	AT5G47390
Achn356431	AT1G74840
Achn216541	AT3G16350
Achn092691	AT4G12350
Achn137671	AT1G74840
Achn198731	AT3G13890
Achn211821	AT2G39880
Achn013291	AT3G09230
Achn211821	AT3G55730
Achn368681	AT4G32730
Achn337181	AT5G02320
Achn351781	AT5G02840
Achn121851	AT1G74840
Achn121951	AT1G74650
Achn102731	AT3G06490
Achn358581	AT4G25560
Achn132371	AT1G63910
Achn217431	AT1G74840
Achn108411	AT3G46130
Achn234881	AT3G28910
Achn297221	AT5G40350
Achn212761	AT2G36890
Achn148821	AT3G13540
Achn228371	AT3G06490
Achn081171	AT5G52260
Achn389471	AT3G27810
Achn363591	AT3G49850
Achn229071	AT5G15310
Achn363591	AT5G67580
Achn307261	AT5G14340
Achn389471	AT5G40350
Achn040631	AT2G37630
Achn031311	AT4G21440
Achn020361	AT2G16720
Achn020361	AT4G34990

Table S7. *MYB* syntenic pairs of kiwifruit and rice.

Kiwifruit	Rice
Achn163941	LOC_Os01g62410
Achn013291	LOC_Os01g63160
Achn036201	LOC_Os01g63460
Achn337181	LOC_Os01g62410
Achn141271	LOC_Os05g37730
Achn108411	LOC_Os11g47460
Achn154591	LOC_Os01g63460
Achn154591	LOC_Os05g37730
Achn020361	LOC_Os09g36730

Table S8. The differentially expressed gene of *AcR2R3-MYB* from the kiwifruit transcriptomic data under drought stress.

Gene_id	readcount_DR	readcount_CKD	log_2FoldChange	pval	padj	significant
Achn173251	32.04309295	2.337128134	3.7772	1.64×10^{-6}	1.38×10^{-5}	TRUE
Achn228371	87.5516779	7.493796485	3.5464	1.73×10^{-6}	1.44×10^{-5}	TRUE
Achn102731	487.4958298	63.94239665	2.9305	9.49×10^{-39}	8.10×10^{-37}	TRUE
Achn031311	239.9189215	42.72556048	2.4894	8.28×10^{-20}	2.44×10^{-18}	TRUE
Achn352391	330.8635929	80.34440746	2.042	1.37×10^{-18}	3.66×10^{-17}	TRUE
Achn345001	141.3400146	35.18180914	2.0063	2.40×10^{-10}	3.24×10^{-9}	TRUE
Achn324811	1037.373376	357.1481473	1.5383	3.10×10^{-17}	7.50×10^{-16}	TRUE
Achn111201	1495.31516	688.4516795	1.119	0.0058164	0.023593	TRUE
Achn337181	237.0491278	136.9678496	0.79135	0.00040059	0.002165	TRUE
Achn121951	1543.927196	979.6714404	0.65623	0.0097843	0.03682	TRUE
Achn368681	1393.034323	1824.422294	−0.38921	0.0080483	0.031231	TRUE
Achn116791	24.86074739	70.5634377	−1.5051	0.00026289	0.001477	TRUE
Achn109411	37.98874878	114.8611216	−1.5962	1.45×10^{-6}	1.23×10^{-5}	TRUE
Achn215991	43.46668042	192.9418787	−2.1502	1.53×10^{-5}	0.00011	TRUE
Achn377521	45.88458416	231.0274558	−2.332	4.14×10^{-18}	1.07×10^{-16}	TRUE
Achn172901	3.440552096	56.69837443	−4.0426	5.77×10^{-11}	8.31×10^{-10}	TRUE
Achn317571	2.37826233	40.7459573	−4.0987	1.85×10^{-8}	2.03×10^{-7}	TRUE
Achn382461	0.935448367	17.68224605	−4.2405	0.0019374	0.008979	TRUE

Table S9. The differentially expressed gene of *AcR2R3-MYB* from the kiwifruit transcriptomic data under high temperature stresses.

Gene_id	readcount_HT	readcount_CKH	log_2FoldChange	pval	padj	significant
Achn121951	837.2821609	72.49329365	3.5298	1.27×10^{-5}	0.000123	TRUE
Achn102731	37.47582507	10.388456	1.851	0.0085518	0.03512	TRUE
Achn109411	292.2492902	108.881127	1.4244	0.006153	0.026781	TRUE
Achn195411	290.9091135	122.6623002	1.2459	0.00045211	0.002896	TRUE
Achn368681	871.8153777	1448.933059	−0.7329	0.0034	0.016269	TRUE
Achn172271	452.9221515	858.6165728	−0.92275	0.0008463	0.004985	TRUE
Achn361141	193.1887498	380.4208597	−0.97759	0.0015907	0.008528	TRUE

Achn211821	43.64464877	99.07649447	−1.1827	0.0079291	0.033018	TRUE
Achn337181	51.33324868	123.9273023	−1.2715	0.003578	0.017007	TRUE
Achn225741	67.59464366	173.3403362	−1.3586	0.00041513	0.002693	TRUE
Achn382461	24.98618387	77.96073489	−1.6416	0.001932	0.010082	TRUE
Achn370001	17.11798434	54.52527537	−1.6714	0.0052087	0.023287	TRUE
Achn012091	22.76871355	84.111322	−1.8852	0.00020963	0.00148	TRUE
Achn322351	325.1519837	1386.451976	−2.0922	2.61×10^{-15}	1.02×10^{-13}	TRUE
Achn294101	126.6095908	658.4426091	−2.3787	3.00×10^{-15}	1.17×10^{-13}	TRUE
Achn223911	24.47499083	155.7921684	−2.6702	2.35×10^{-9}	4.58×10^{-8}	TRUE
Achn098041	4.959936561	51.48503253	−3.3758	2.20×10^{-5}	0.000201	TRUE
Achn143561	37.28932402	597.5007258	−4.0021	1.29×10^{-24}	1.15×10^{-22}	TRUE
Achn116791	1.3677824	138.1337768	−6.6581	4.19×10^{-19}	2.43×10^{-17}	TRUE

Table S10. Cis-acting element analysis of *AcMYBs* promoter in kiwifruit.

Gene symbol	Cis-acting element	sequence	Number	Induced response
MYB60	GARE-motif	TCTGTTG	1	gibberellin
（Achn121951）	CGTCA-motif	CGTCA	1	MeJA
	TGACG-motif	TGACG	1	MeJA
	TGA-element	AACGAC	2	auxin
	ABRE	ACGTG	1	abscisic acid
	ARE	AAACCA	3	anaerobic induction
	LTR	CCGAAA	3	low-temperature
	MBS	CAACTG	1	drought
	MRE	AACCTAA	1	light
MYB2	TGACG-motif	TGACG	1	MeJA
（Achn324811）	CGTCA-motif	CGTCA	1	MeJA
	TGA-element	AACGAC	1	auxin
	ABRE	AACCCGG	1	abscisic acid
	ABRE	ACGTG	3	abscisic acid
	TC-rich repeats	ATTCTCTAAC	1	defense and stress
	LTR	CCGAAA	1	low-temperature
	ARE	AAACCA	3	anaerobic induction
MYB102	P-box	CCTTTTG	1	gibberellin
（Achn173251）	TGACG-motif	TGACG	2	MeJA
	CGTCA-motif	CGTCA	2	MeJA
	AuxRR-core	GGTCCAT	1	auxin
	ABRE	ACGTG	2	abscisic acid
	ABRE	GACACGTGGC	1	abscisic acid
	MBS	CAACTG		drought
	ARE	AAACCA	2	anaerobic induction
MYB61	TCA-element	CCATCTTTTT		salicylic acid
（Achn215991）	ABRE	ACGTG	1	abscisic acid
	ARE	AAACCA	2	anaerobic induction

	MRE	AACCTAA	2	light
